# Early neurovascular uncoupling in the brain during community acquired pneumonia

**DOI:** 10.1186/cc11310

**Published:** 2012-04-20

**Authors:** Bernhard Rosengarten, Dennis Krekel, Stefan Kuhnert, Richard Schulz

**Affiliations:** 1 Department of Neurology, Justus-Liebig University of Giessen, Klinikstrasse 33, 35392 Giessen, Germany; 2 Department of Internal Medicine, Medical Clinic II, Justus-Liebig University of Giessen, Klinikstrasse 33, 35392 Giessen, Germany

## Abstract

**Introduction:**

Sepsis leads to microcirculatory dysfunction and therefore a disturbed neurovascular coupling in the brain. To investigate if the dysfunction is also present in less severe inflammatory diseases we studied the neurovascular coupling in patients suffering from community acquired pneumonia.

**Methods:**

Patients were investigated in the acute phase of pneumonia and after recovery. The neurovascular coupling was investigated with a simultaneous electroencephalogram (EEG)-Doppler technique applying a visual stimulation paradigm. Resting EEG frequencies, visual evoked potentials as well as resting and stimulated hemodynamic responses were obtained. Disease severity was characterized by laboratory and cognitive parameters as well as related scoring systems. Data were compared to a control group.

**Results:**

Whereas visually evoked potentials (VEP) remained stable a significant slowing and therefore uncoupling of the hemodynamic responses were found in the acute phase of pneumonia (Rate time: control group: 3.6 ± 2.5 vs. acute pneumonia: 1.6 ± 2.4 s; *P *< 0.0005). In the initial investigation, patients who deteriorated showed a decreased hemodynamic response as compared with those who recovered (gain: recovered: 15% ± 4% vs. deteriorated: 9% ± 3%, *P *< 0.05; control: 14% ± 5%). After recovery the coupling normalized.

**Conclusions:**

Our study underlines the role of an early microcirculatory dysfunction in inflammatory syndromes that become evident in pre-septic conditions with a gradual decline according to disease severity.

## Introduction

Modern sepsis concepts and early goal-directed therapies increasingly focus on the microcirculation and its integrity in inflammatory syndromes [1-5]. A microcirculatory dysfunction was found in many organs such as gut, heart, lung, and brain [6-8]. Nowadays, the occurrence of a microcirculatory dysfunction is regarded as an important motor of sepsis-associated organ dysfunction [1-4,8]. Clinically, the microcirculatory dysfunction correlates with an increased mortality rate and worse outcome [9-12].

The brain lacks relevant oxygen or energy stores and therefore is highly dependent on an adequate blood supply [13]. The neurovascular coupling (NC) is a brain-intrinsic vasoregulatory mechanism that adapts the local cerebral blood flow in accordance with the metabolic needs of active neurons. Neurovascular uncoupling due to microvascular dysfunction results in an inadequate blood supply of active neurons and is assumed to be a relevant factor of sepsis-associated encephalopathy [14-18].

Since most investigations regarding the brain microcirculation were performed in patients with sepsis, the question of whether a microvascular dysfunction is even present in less severe inflammatory syndromes arises. Community-acquired pneumonia (CAP) is a frequent and important inflammatory disease with a high risk to run into a sepsis syndrome [12,19]. We aimed at investigating patients with CAP in the acute phase and after recovery and compared data with a control group of similar age. Disease severity was specified by different intensive care and pneumonia scoring systems. By applying visual stimuli, a simultaneous electroencephalogram (EEG)-Doppler technique allowed investigation of the NC by evaluating visually evoked potentials (VEPs) together with the resultant evoked flow velocity responses. Cognitive integrity was screened by the Glasgow Coma Scale (GCS) and Intensive Care Delirium Screening Checklist (ICDSC).

## Materials and methods

### Inclusion criteria and examination protocols

The local ethics committee of the Justus-Liebig University of Giessen approved this non-interventional study, which was performed in accordance with the ethical standards of the Declaration of Helsinki (1975). All subjects were informed about the study and gave written informed consent to participate. The study compared a patient group suffering from CAP with a healthy control group of similar age. Consecutively, patients admitted to the internal department for stationary CAP therapy were included. Excluded were patients with disturbances of the visual system, with previous stroke, or with significant stenoses of the posterior cerebral circulation as examined by transcranial Doppler. Patients with malignancies or pulmonary, renal, cardiac, or hepatic organ failure were also excluded. For technical reasons, patients with an insufficient temporal bone window hampering the Doppler recording were disregarded. A first examination was performed on the day following admittance when the therapeutic regimen was fixed and the patients were under antibiotic medication according to the therapeutic guidelines of CAP [19]. In all patients, fever was treated with antipyretics to normalize body temperature. A second examination was undertaken after a minimum of 7 days when patients already recovered but were still under the same medication.

The diagnostic workup included a clinical examination and technical and laboratory tests. Clinical examination included auscultation of the chest (presence of inspiratory crackles) and determination of the respiratory rate and arterial blood pressure (measured with a cuff technique on the upper arm). Laboratory tests included leukocyte numbers, C-reactive protein (CRP) level, and blood gas analysis and were performed on the day of the EEG-Doppler test. Finally, a chest x-ray was done to detect pulmonary infiltrates. Vascular risk factors that could interfere with vascular function - such as body mass index, smoking habit (measured in pack-years), hyperlipidemia, hypertension, coronary artery disease, and diabetes mellitus - were also obtained. Also, the medication with angiotensin-converting enzyme or angiotensin receptor blocker, diuretics, statins, calcium antagonists, glucocorticoids, beta-blocker, beta-mimetics, theophylline, platelet aggregation inhibitors, nitrates, prostaglandins, or sympathomimetics was obtained.

To score the disease severity, we chose the Acute Physiology and Chronic Health Evaluation II (APACHE II) score [20]. To assess the severity of the CAP, we used the pneumonia severity index (PSI) and graded the patients in a risk score ranging from grade I to V [21]. Also, the CURB-65 - confusion of new onset (defined as an abbreviated mental test score of 8 or less), urea of greater than 7 mmol/L (19 mg/dL), respiratory rate of 30 breaths per minute or greater, systolic blood pressure of less than 90 mm Hg or diastolic blood pressure of 60 mm Hg or less, and age of at least 65 years - was obtained [22]. To specify the degree of dyspnea of the patients, we chose the American Thoracic Society Scale (ATS) [23]. To gather cerebral integrity, we obtained the GCS and ICDSC scores [24].

### Stimulation paradigm

We used, as a stimulation paradigm, a modified checkerboard test in which the volunteers focused on a spot in the center of a 21-inch liquid crystal display stimulation monitor. The monitor had a picture repetition time of 5 milliseconds (Iiyama Corporation, Kitaowaribe, Nagano, Japan). The volunteers were sitting quietly at 1 meter from the monitor. The stimulation protocol consisted of 10 cycles, each with a resting phase of 20 seconds and a stimulating phase of 40 seconds. During resting periods, volunteers were instructed to close their eyes; during stimulation phases, they had to look at the screen. Changes between phases were signaled acoustically by a tone. The stimuli consisted of black and white pictures that alternated with their negatives to induce contrast-based visually evoked responses. The contrast between white and black areas was calculated to a c value of 92%. The present stimulation paradigm was much more comfortable for the volunteers as compared with a classic checkerboard pattern [25]. The flickering frequency was set to 1 Hz, meaning that a picture was shown for 500 milliseconds followed by its negative for 500 milliseconds. During the 40-second stimulation phase, 80 reversals were performed.

### Doppler recording

For the Doppler recording, two 2-MHz probes were mounted on an individually fitted head-band. In all cases, the P2 segment of both posterior cerebral arteries was insonated. Peak systolic blood flow velocities were recorded by using a Multidop T2 Doppler device (DWL, Singen, Germany). The reason for evaluating systolic velocities is that the index is less prone to Doppler artefacts. The beat-to-beat intervals of cerebral blood flow velocity were interpolated in a linear fashion with a 'virtual' time resolution of 50 milliseconds. To ensure independence from the insonation angle and to allow comparisons between volunteers, absolute values were transformed into relative changes of cerebral blood flow velocity in relation to the baseline. The baseline was calculated from the blood flow velocity averaged over a time span of 5 seconds before the beginning of the stimulation phase and set to zero. The method and algorithm for analyzing the data sets in terms of a control system are described in detail elsewhere [26]. The following parameters were specified: K represents the gain, Tv the rate time, ω the undampened natural angular frequency (natural frequency), and ζ the attenuation parameter of the system.

The parameters describe the dynamic features of the NC with an initial rapid upstroke of flow velocity, overshoot, and succeeding decay to a stable flow velocity level above baseline. The gain represents the flow velocity difference between conditions of rest and activation under stable hemodynamic conditions. The rate time indicates the initial steepness of the flow velocity increase. Natural frequency and the attenuation describe oscillation features of the system. The natural frequency is assumed to represent the tonus and the speed of the system, whereas the attenuation describes dampening features such as elastic properties of the vessel wall.

### Electroencephalogram recording

From a 16-channel digital EEG (Schwarzer, Munich, Germany), six channels were used for electrical VEP recording of the field potential. Signals were recorded from Fp1 and Fp2, from O1 and O2, and from the ears, A1 and A2. The reference electrode was set to Fz. The electrodes were placed according to the positions specified in the international 10-20 system. Also, an electrocardiogram was obtained with electrodes placed on both forearms and digitized with the EEG data. Data were sampled at a rate of 1 kHz. An online band-pass filter was applied with a high-pass filter setting of 0.3 Hz and a low-pass filter setting of 70 Hz. Functionally evoked electrical activity changes were recorded from the scalp by means of silver chloride electrodes fixed by an EEG headset. Resistance was kept below 5 kΩ. The resting EEG activity was Fourier-transformed to calculate the percentage distribution of the typical EEG bands. The frequencies below 4 Hz were assumed to be in the range of the delta band, frequencies between 4 and 7 Hz as theta, between 8 and 12 Hz as alpha, and between 13 and 30 Hz as beta waves. Calculations were performed by the EEG device. VEPs were calculated offline from the EEG signals. To quantify the typical peaks from the VEP waveform, the data from 80 stimulations during a 40-second stimulation phase were averaged. Analysis of typical peaks (N75 and P100) was performed, and amplitude differences were calculated [25].

### Statistical analysis

Comparing the first and second measurements in patients with CAP together with the controls, the clinical as well as VEP and Doppler data were statistically analyzed with a one-way analysis of variance (ANOVA). Also, the data between the recovery/deterioration groups were analyzed by an ANOVA. In case of significance, a paired comparison with the Scheffé *post hoc *test was performed. Normal distribution was tested with an F test. Alternatively, a non-parametric Kruskal-Wallis test was used for the tree group comparison, whereas a Mann-Whitney *U *test was used to compare the data comparing the recovery/deterioration groups. The level of significance was set to a *P *value of less than 0.05.

## Results

The study included 50 patients with CAP; in 43 of these patients, a complete data set of both electrical and hemodynamic recordings was eligible for evaluation. Thirty patients could be examined after recovery, and 24 of these patients had a complete data set. The second measurement was performed 8.5 ± 1.7 days after the first examination. Seven patients deteriorated with sepsis and septic shock. Six patients could not be followed up. Thirty healthy volunteers in a similar age range served as controls. The group characteristics, together with clinical profiles, are presented in Table 1. Patients presented at admission with a PSI score of 99 ± 27, which corresponds to a risk score of IV, and with therapy improved significantly to 86 ± 24 (*P *< 0.05), which corresponds to a risk score of III. The CURB-65 score was around 1.5 ± 0.9 at admission and improved non-significantly to 1.3 ± 0.9 under recovery. The APACHE II score was initially 13 ± 4 and with therapy improved to 11 ± 4 (*P *< 0.05). The ATS score indicated, on admission, a nearly severe dyspnea, which with recovery significantly improved to a moderate dyspnea level (2.9 ± 0.8 to 1.9 ± 0.7; *P *< 0.0001). The GCS score did not significantly differ between the first and second examinations, whereas the ICDSC score showed a strong improvement (3 ± 1 to 1 ± 1; *P *< 0.0001). From the physiological and laboratory parameters, a significant lowering of the CRP level (95 ± 22 to 23 ± 23; *P *< 0.0001) and improvement of the oxygen saturation (93 ± 4 to 96 ± 4; *P *< 0.01) were found, whereas leukocyte count, temperature, and pH levels did not differ between first and second examinations. In both pneumonia groups, the EEG showed a significant drop in the relative frequency of the alpha band and increases in the theta and beta bands (Table 1). This was accompanied by a slowing of the peak frequency in the alpha band and fastening of that in the theta band. In comparison with controls, the power in the theta band was significantly higher in both pneumonia groups.

**Table 1:** Group data for vascular risk factors, medication, and resting EEG data together with ANOVA statistical results

	Acute pneumonia (A)	Recovery from pneumonia (R)	Control (C)	ANOVA
Age, years	67 ± 13	65 ± 11	66 ± 8	NS
Number	43	24	30	-
Female/male ratio	30/13	17/7	16/14	-
RR mean, mm Hg	83 ± 12	86 ± 12	82 ± 14	NS
Body mass index	24 ± 6	28 ± 6	24 ± 6	NS
Smoker, pack-years	46 ± 8	31 ± 13	-	NS
Diabetes, percentage	17	14	-	NS
Coronary artery disease, percentage	44	29	-	NS
Leukocytes, G/L	11 ± 5	10 ± 4	N.O.	NS
CRP, mg/dL	95 ± 22	23 ± 23	N.O.	*P *< 0.0001
Temperature,°C	36.7 ± 0.6	36.3 ± 0.3	N.O.	NS
pH	7.37 ± 0.06	7.40 ± 0.03	N.O.	NS
Hkt, percentage	38 ± 6	38 ± 5	N.O.	NS
sO_2 _percentage	93 ± 4	96 ± 4	N.O.	*P *< 0.01
ATS score	2.9 ± 0.8	1.9 ± 0.7	N.O.	*P *< 0.0001
PSI score	99 ± 27	86 ± 24	N.O.	*P *< 0.05
CURB-65 score	1.5 ± 0.9	1.3 ± 0.9	N.O.	NS
APACHE II score	13 ± 4	11 ± 4	N.O.	*P *< 0.05
GCS score	15 ± 0.5	15 ± 0.4	N.O.	NS
ICDSC score	3 ± 1	1 ± 1	N.O.	*P *< 0.0001
EEG μV^2 ^delta	82 ± 71	81 ± 58	98 ± 68	NS
EEG μV^2 ^theta	58 ± 62	56 ± 52	26 ± 12	C-A: *P *< 0.01; C-R: *P *< 0.01
EEG μV^2 ^alpha	118 ± 126	126 ± 98	151 ± 128	NS
EEG μV^2 ^beta	40 ± 25	44 ± 24	31 ± 12	NS
EEG percentage delta	30 ± 15	27 ± 13	31 ± 16	NS
EEG percentage theta	19 ± 12	22 ± 11	10 ± 3	C-A: *P *< 0.001; C-R: *P *< 0.0001
EEG percentage alpha	35 ± 17	35 ± 13	47 ± 20	C-A: *P *< 0.01; C-R: *P *< 0.05
EEG percentage beta	16 ± 7	16 ± 9	12 ± 5	C-A: *P *< 0.05
EEG peak Hz delta	1.2 ± 0.3	1.2 ± 0.4	1.2 ± 0.3	NS
EEG peak Hz theta	6.6 ± 0.9	7.1 ± 0.7	6 ± 0.8	C-A: *P *< 0.001; C-R: *P *< 0.0001 A-R: *P *< 0.05
EEG peak Hz alpha	9.4 ± 0.9	9.1 ± 0.8	11 ± 0.8	C-A: *P *< 0.0001; C-R: *P *< 0.0001
EEG peak Hz beta	15.9 ± 2	15.6 ± 2.1	15.9 ± 2.2	NS

Data are presented as mean ± standard deviation unless otherwise indicated. ANOVA, analysis of variance; APACHE II, Acute Physiology and Chronic Health Evaluation II; ATS, American Thoracic Society Scale; CRP, C-reactive protein; CURB-65, confusion of new onset (defined as an abbreviated mental test score of 8 or less), urea of greater than 7 mmol/L (19 mg/dL), respiratory rate of 30 breaths per minute or greater, systolic blood pressure of less than 90 mm Hg or diastolic blood pressure of 60 mm Hg or less, and age of at least 65 years; EEG, electroencephalogram; GCS, Glasgow Coma Scale; Hkt, hematocrit; ICDSC, Intensive Care Delirium Screening Checklist; N.O., nitric oxide; NS, not significant; PSI, pneumonia severity index; RR, blood pressure; sO_2_, blood oxygen saturation.

**Figure 1:**
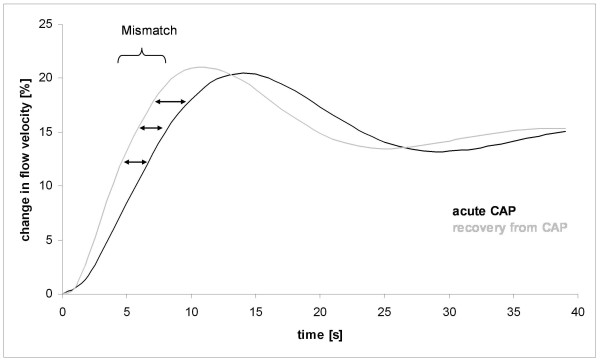
**The modeled hemodynamic flow velocity responses due to visual stimulation are shown as relative changes to resting flow velocity**. The resting flow velocity level was set to zero. During the acute phase of community-acquired pneumonia (CAP), the initial hemodynamic responses were delayed, resulting in a lag of blood flow adjustment of about 3 to 4 seconds as compared with the hemodynamic responses after recovery from CAP. After compensation of the initial mismatch, flow velocity levels did not differ at the end of the stabilization phase.

Table 2 shows the results from the NC test. There was a significant difference between groups in regard to the initial dynamic phase of vasoregulation. The rate time parameter describes the initial speed of flow velocity adaptation. The significantly smaller parameter in the acute-phase group indicates a slower upstroke and therefore a delayed cerebral blood flow adaptation (Figure 1). With recovery from pneumonia, a normalization of the parameter was seen. The VEP, the resting flow velocity level, and the other hemodynamic parameter did not differ between the three groups.

**Table 2:** Neurovascular function test

	VEP N1-P1, μV	Resting flow velocity, cm/second	Gain, percentage	Natural frequency, 1/second	Attenuation	Rate time, seconds
Control group	7.6 ± 2.7	45 ± 10	14 ± 5	0.21 ± 0.05	0.49 ± 0.18	3.6 ± 2.5

Acute pneumonia	7.7 ± 3.2	47 ± 13	15 ± 8	0.21 ± 0.08	0.43 ± 0.18	1.6 ± 2.4^a^

Recovery from pneumonia	7.2 ± 2.3	45 ± 11	15 ± 6	0.23 ± 0.06	0.50 ± 0.35	3.4 ± 2.4

ANOVA	NS	NS	NS	NS	NS	*P *< 0.0005

Except for analysis of variance (ANOVA), data are presented as mean ± standard deviation. ^a^Indicates *P *< 0.01 result of the Scheffé *post hoc *test. NS, not significant; VEP, visually evoked potential.

Although the number of patients who deteriorated after the first examination is limited, a comparison with the initial data of those patients who recovered revealed a significantly higher PSI score, a trend to a higher APACHE II score, and similar ATS and CURB-65 scores (Table 3). The GCS score was significantly lower whereas the ICDSC score could not discriminate between groups. Interestingly, all patients who had a bad outcome had a history of coronary artery disease. This finding was significant compared with the group that recovered (*P *< 0.05). Another significant finding pointing to a worse vascular functioning was the gain parameter, which was lower in the patient group with worse outcome. Other indicators for a deterioration were trends to a lower pH level and a lower body temperature (both *P *= 0.07) (Table 3). No patient took nitrates, prostaglandins, or sympathomimetics. Analysis of the medication revealed no significant difference between the groups.

**Table 3:** Comparison of initial patient data grouped according to improvement or deterioration of clinical conditions

Acute-phase examination	Patients who recovered (*n *= 24)	Patients who deteriorated (*n *= 7)	Statistics
RR mean, mm Hg	83 ± 13	84 ± 12	NS

Body mass index	28 ± 6	24 ± 4	NS

Smoking, pack-years	31 ± 13	57 ± 20	NS

Diabetes, percentage	14	33	NS

Coronary artery disease, percentage	29	100	*P *< 0.05

pH	7.37 ± 0.06	7.33 ± 0.04	*P *= 0.07

Leukocytes, g/L	11 ± 5	11 ± 5	NS

Temperature,°C	36.8 ± 0.6	36.3 ± 0.3	*P *= 0.07

ATS score	3 ± 0.8	3 ± 0.8	NS

PSI score	101 ± 23	125 ± 13	*P *< 0.05

CURB-65 score	1.6 ± 0.9	2.0 ± 0.6	NS

APACHE II score	13 ± 4	15 ± 2	*P *= 0.06

GCS score	15 ± 0.3	14 ± 0.8	*P *< 0.05

ICDSC score	3 ± 1	3 ± 1	NS

ACE/AT inhibitors, percentage	36	66	NS

Calcium antagonists, percentage	18	17	NS

Beta-blocker, percentage	32	66	NS

Diuretics, percentage	77	100	NS

Statins, percentage	14	33	NS

Thrombo inhibitors, percentage	32	66	NS

Glucocorticoids, percentage	41	33	NS

β2 mimetics, percentage	64	83	NS

Theophylline, percentage	23	17	NS

EEG μV^2 ^delta	83 ± 74	68 ± 43	NS

EEG μV^2 ^theta	60 ± 64	43 ± 41	NS

EEG μV^2 ^alpha	122 ± 131	77 ± 65	NS

EEG μV^2 ^beta	42 ± 27	29 ± 15	NS

EEG percentage delta	30 ± 16	31 ± 11	NS

EEG percentage theta	19 ± 12	19 ± 14	NS

EEG percentage alpha	35 ± 17	33 ± 17	NS

EEG percentage beta	16 ± 7	17 ± 9	NS

EEG peak Hz delta	1.2 ± 0.4	1.3 ± 0.3	NS

EEG peak Hz theta	6.6 ± 0.9	6.3 ± 1	NS

EEG peak Hz alpha	9.3 ± 0.9	9.7 ± 0.9	NS

EEG peak Hz beta	15 ± 2	16.4 ± 2.5	NS

Visual evoked potential, μV	7.4 ± 2.3	7.1 ± 2.8	NS

Resting flow velocity, cm/second	47 ± 14	48 ± 17	NS

Gain, percentage	15 ± 4	9 ± 3	*P *< 0.05

Natural frequency, 1/second	0.21 ± 0.03	0.22 ± 0.08	NS

Attenuation	0.41 ± 0.19	0.31 ± 0.27	NS

Rate time, seconds	1.6 ± 1.3	0.9 ± 0.3	NS

Data are presented as mean ± standard deviation unless indicated otherwise. ACE, angiotensin-converting enzyme; APACHE II, Acute Physiology and Chronic Health Evaluation II; AT, angiotensin; ATS, American Thoracic Society Scale; CURB-65, confusion of new onset (defined as an abbreviated mental test score of 8 or less), urea of greater than 7 mmol/L (19 mg/dL), respiratory rate of 30 breaths per minute or greater, systolic blood pressure of less than 90 mm Hg or diastolic blood pressure of 60 mm Hg or less, and age of at least 65 years; EEG, electroencephalogram; GCS, Glasgow Coma Scale; ICDSC, Intensive Care Delirium Screening Checklist; NS, not significant; PSI, pneumonia severity index; RR, blood pressure.

## Discussion

Patients on admission presented with a grade IV CAP according to the PSI score and were treated as high-risk patients on an internal ward. The CURB-65 score was about 1.5, indicating a lethality risk of between 3% and 13% [23]. The APACHE II score was initially around 13 points, indicating a lethality of 15% [27]. The ATS level indicated a severe dyspnea confirmed by the oxygen saturation of 93% ± 4%. Besides showing the typical clinical and laboratory parameters, patients showed a typical slowing of EEG, mainly into the theta band [28]. Owing to the sensitivity of the ICDSC, the test could differentiate between the acute phase and the recovery whereas the GCS was insensitive [24]. However, in the acute phase, the GCS could give additional information since it could discriminate between the group that recovered and that which deteriorated, whereas the ICDSC was positive in both cases.

The new finding was that the patients with CAP presented with a neurovascular uncoupling in the acute inflammatory phase. This was concluded from dissociation of electrical and hemodynamic data. A significantly delayed hemodynamic adaptation to cortical activation (rate time) occurred despite unchanged VEP amplitudes and latencies. A decreased rate time parameter indicates a reduced speed of the NC. A change from rest to activation results in an increase in neuronal activity and thus metabolic needs in the millisecond range, whereas the hemodynamic adaptation works in the second range. The different response times lead to an initial perfusion mismatch that lasts about 8 to 10 seconds even under physiological conditions but is significantly prolonged in the acute phase of patients with CAP. Owing to a continuously changing brain activity, the slowing of the NC leads, on average, to a reduced cerebral perfusion [29]. Patients who deteriorated showed a significantly decreased gain parameter that further reduced adequate blood supply of active neurons. Since the PSI score and the other disease parameters were also increased in that group, it appears that the drop in the gain parameter might be related to the stronger inflammatory process. The present results mimic animal sepsis experiments in which a vascular uncoupling preceded changes in evoked potential amplitudes and in which the uncoupling was dependent on the severity of inflammatory induction [14]. Although the findings were discrete, the uncoupling might have clinical relevance since it was shown that a cerebral perfusion mismatch of only 10% results in protein synthesis disturbances and dysfunction of neurons [8]. Though indicative of a disturbed NC, the matter with the reduced gain parameter and its relation to the CAP has to be addressed in more detail. Since we do not have data from the patients in the group that deteriorated under non-inflammatory conditions, the high frequency of coronary artery disease in this group gains relevance. Each patient in the deteriorating group had a history of coronary artery disease, whereas in the recovery group, this was only in 29% of cases. It is known that the severity of the coronary artery disease is correlated with a decreased gain parameter [30]. However, patients with previous vascular diseases are known to be more prone to a more severe sepsis syndrome and worse outcome [1-3]. Therefore, the possibility exists that the reduced gain parameter was caused by a more severe atherosclerosis rather than by a more pronounced inflammation. Anyway, both explanations point to a pivotal role of the vascular system and microcirculation in inflammatory syndromes.

At early phases of an inflammatory syndrome, the microcirculatory dysfunction could not be concluded from systemic hemodynamic indices such as blood pressure or overall organ perfusion, because their correlation is very loose [8,9]. We also did not find a significant difference between groups in regard to the blood pressure or cerebral resting flow velocities. Only with functional activation experiments were we able to unmask a microcirculatory dysfunction in the visual cortex.

The present technique using visual stimuli has another advantage in comparison with other studies, which have used acetazolamide or carbogene gas to assess microvascular reactivity in patients with sepsis [31,32]. Both agents induce a shift in the interstitial pH level, which is the trigger for the microvascular reaction. Since the sepsis itself leads to considerable pH changes, this might bias test results [33,34].

A limitation of the present study might be the relatively small patient number, so the present findings have to be corroborated in studies with a bigger sample size. However, owing to the simultaneous investigation of electrical and hemodynamic responses, the technique has a higher sensitivity in comparison with other studies in which serial investigations have been performed. Furthermore, a clinically feasible tool should allow individual examinations. The present EEG-Doppler approach meets these recommendations and its sensitivity has been demonstrated in other clinical investigations.

## Conclusions

It appears from the present study that the occurrence of a microcirculatory dysfunction seems to gradually depend on the severity of the inflammatory process. Since patients with vascular diseases have a smaller compensative range, they might be at higher risk, possibly explaining their worse outcome in sepsis syndromes. Our data support modern guidelines that recommend a microvascular monitoring of patients in early stages of inflammatory syndromes.

## Key messages

• Neurovascular uncoupling occurs early in patients with community-acquired pneumonia.

• Firstly, neurovascular uncoupling results in a slowing of the initial hemodynamic blood flow adaptation (rate time parameter) whereas the level of the blood flow velocity change under stabilized hemodynamic conditions remains constant (gain parameter).

• Secondly, a decline in the gain parameter occurs in cases that are more severe.

• Our data support concepts of a critical role of a microcirculatory dysfunction as a motor of sepsis-associated encephalopathy.

## Abbreviations

ANOVA: analysis of variance; APACHE II: Acute Physiology and Chronic Health Evaluation II; ATS: American Thoracic Society Scale; CAP: community-acquired pneumonia; CRP: C-reactive protein; CURB-65: confusion of new onset (defined as an abbreviated mental test score of 8 or less), urea of greater than 7 mmol/L (19 mg/dL), respiratory rate of 30 breaths per minute or greater, systolic blood pressure of less than 90 mm Hg or diastolic blood pressure of 60 mm Hg or less, and age of at least 65 years; EEG: electroencephalogram; GCS: Glasgow Coma Scale; ICDSC: Intensive Care Delirium Screening Checklist; NC: neurovascular coupling; PSI: pneumonia severity index; VEP: visually evoked potential.

## Competing interests

The authors declare that they have no competing interests.

## Authors' contributions

BR and DK helped to plan and perform the study, evaluate and discuss data, and write the manuscript. SK and RS helped to screen for patients, evaluate the medical records, perform the laboratory investigations, evaluate and discuss data, and write the manuscript. All authors read and approved the final manuscript.
